# The Impact of a Breast Cancer Diagnosis on the Social Interaction Patterns of Young Omani Women: A Qualitative Study Approach

**DOI:** 10.3390/curroncol31120589

**Published:** 2024-12-16

**Authors:** Mohammed Al-Azri, Zayana AL-Kiyumi, Khalid Al-Bimani, Huda Al-Awaisi

**Affiliations:** 1 Department of Family Medicine and Public Health, College of Medicine and Health Sciences, Sultan Qaboos University, Muscat 123, Oman; 2 Sultan Qaboos Comprehensive Cancer Care and Research Centre, University Medical City, Muscat 123, Oman; z.alkiyumi@cccrc.gov.om (Z.A.-K.); k.albaimani@cccrc.gov.om (K.A.-B.); h.alawaisi@cccrc.gov.om (H.A.-A.)

**Keywords:** breast cancer, survivors, social interactions, emotions, qualitative research, Oman

## Abstract

Background and Aim: Young women diagnosed with breast cancer (BC) face considerable psychological and emotional distress, impacting their interactions with themselves, their families, and the wider community. This study sought to explore the interaction patterns of young Omani BC survivors following their diagnosis and during treatment. Materials and Methods: Semi-structured individual interviews were conducted with 11 Omani women diagnosed with BC, recruited from the Sultan Qaboos Comprehensive Cancer Care and Research Centre in Muscat, Oman. Participants were selected using purposive sampling to target Omani BC survivors aged under 45 years, with one to five years of survivorship post-diagnosis. Framework analysis was employed to analyse the qualitative data. Results: Six key types of interactions with various groups were identified: self, children, spouses, family, friends, and society. While many BC survivors demonstrated resilience through their strong faith, viewing the illness as part of a divine plan, others experienced diminished confidence and social withdrawal due to body image issues. Interactions with children centred on maintaining normalcy, while spouses typically provided emotional support despite challenges with intimacy. Family and friends offered crucial support, although concerns regarding societal stigma led some women to conceal their diagnosis. Conclusions: Participants in this study demonstrated a strong sense of acceptance of their cancer diagnosis as a result of their faith, viewing it as part of a divine plan. Their belief in divine guidance, paired with optimism about the available treatments, fostered resilience, allowing them to maintain a calm and hopeful outlook during their journey of treatment and recovery. However, some participants experienced a decline in self-confidence, particularly after treatment. This affected their willingness to socialise and interact with others, leading to introversion and a marked withdrawal from social interactions, often related to changed self-perception or fear of judgment following changes in appearance.

## 1. Introduction

Breast cancer (BC) is the most commonly diagnosed cancer worldwide, affecting 2.3 million women in 2020 and causing 685,000 deaths [1]. In the Eastern Mediterranean region, BC is the leading cause of death and disability among women; moreover, the incidence of this type of cancer continues to rise as a result of increased life expectancy, urbanisation, and the adoption of Westernised lifestyles [2]. In Oman, a country in the Arabian Peninsula, BC accounts for 24.5% of all cancers in women and 12.8% of all malignancies overall [3]. The median age upon diagnosis is 49 years, with most Omani women presenting at advanced stages (i.e., stages III or IV) and a low 5-year survival rate of 63% [4]. Despite this, as a result of population increase, the number of BC survivors, particularly among young women, continues to grow [5].

A diagnosis of BC often triggers significant changes in self-perception and social roles and expectations, putting women at risk of psychological distress, including symptoms of anxiety and depression [6,7]. Additionally, BC survivors are prone to a range of psychological morbidities, such as fatigue, negative thoughts, suicidal ideation, fear of death, loneliness, and concerns regarding sexual and body image, all of which may shape perceptions of their quality of life [6]. Moreover, these perceptions are further influenced by challenges to their psychosocial and cultural belief systems, impacting their overall prognosis [7].

Young BC survivors in particular face unique challenges compared to older women, including concerns about fertility, career development, poorer survival outcomes, and evolving familial roles [8]. Additionally, treatments like mastectomy and chemotherapy, often used to treat young women with large tumours or aggressive forms of BC, can have both short- and long-term effects on quality of life [9]. Previous research has shown that Omani women diagnosed with BC express concerns regarding potential disfigurement and loss of femininity as a result of changes to their appearance due to hair loss and other surgical and treatment side-effects [10].

Women diagnosed with breast cancer often face issues such as stigma, societal judgment, and challenges in public acceptance, which can affect their social interactions and emotional well-being [6]. Studies highlight the importance of social support and networks, as they play a critical role in providing emotional and practical assistance, fostering resilience, and improving coping strategies [7]. Effective coping mechanisms include reliance on supportive relationships, active confrontation of stress, and fostering optimism. These elements significantly contribute to better health-related quality of life and psychological resilience [9].

Furthermore, a BC diagnosis can also significantly impact family dynamics, potentially shifting roles and responsibilities within the household [10]. Young survivors may grapple with feelings of guilt or burden, while family members may struggle with uncertainty about how best to provide support [8]. Family members themselves may also experience diminished quality of life when a loved one is diagnosed with BC, underscoring the importance of family resilience to both survivors and caregivers [11]. Resilience in BC survivors can help them to manage daily stressors, fostering positive balance in family functioning and positively influencing their cancer trajectory [12]. Families that successfully adapt to the cancer crisis tend to exhibit cohesive patterns of engagement and find new ways to restore family balance. Cultural factors further shape interactions between survivors and family members during this response process [13].

Thus, the resilience of both BC survivors and their families can ease the burden on caregivers and promote individual resilience in survivors [13]. However, few studies have examined characteristics of family resilience in the context of cancer, especially in young BC survivors [14]. In Oman, where traditional and Western influences often conflict, these family dynamics may present unique characteristics. In Oman, as in other Middle Eastern countries, significant societal stigma surrounding cancer can affect how families interact with survivors, potentially leading to silence, isolation, and avoidance of the topic [10]. Addressing this stigma is crucial for enhancing open communication and support within families. Given the central role of family in supporting young Omani BC survivors, understanding the availability of such support systems and how they can be strengthened is crucial for improving the well-being of survivors [15]. Therefore, this research aimed to explore the interaction patterns of young Omani BC survivors following diagnosis.

## 2. Materials and Methods

### 2.1. Study Setting

The Sultan Qaboos Comprehensive Cancer Care and Research Centre (SQCCCRC), University Medical City, is a major oncological referral centre in Muscat, the capital of Oman. It provides a holistic approach to cancer treatment, incorporating research initiatives and educational resources. Women diagnosed with BC across different regions of Oman are often referred to SQCCCRC, University Medical City, for surgical and oncological treatment [16].

### 2.2. Sampling Strategy

Purposeful sampling, commonly used in qualitative research to target information-rich cases related to the phenomenon of interest [17], was employed to select participants based on specific characteristics relevant to the research question. Cut-off ages for defining young BC survivors vary, with thresholds set at 35, 40, 45, or 50 years across different populations [17,18]. For the purposes of this study, we recruited participants aged 45 years or younger. As such, adult Omani BC survivors aged under 45 years, with 1 to 5 years of survivorship and no prior history of BC, were recruited. Women were approached while attending outpatient appointments and day care visits or while admitted to the oncology wards. Those who were in obvious distress or pain were excluded from the study. Participation was entirely voluntary in nature, with written informed consent obtained from each participant after explaining the purpose of the study.

### 2.3. Semi-Structured Interviews and Topic Guide

Qualitative research aims to provide a comprehensive summary of participants’ experiences and perceptions [19]. To achieve this objective, semi-structured, face-to-face interviews were conducted with each of the selected participants by the second author, a female oncology nurse experienced in qualitative research [20]. Semi-structured interviews were chosen for data collection because they allow the flexibility to probe deeper into relevant topics that emerge during the conversation. This adaptability enables researchers to explore unexpected themes or insights that align with the study’s objectives [20].

Individual interviews took place in private rooms at the outpatient clinic, day care unit, or oncology ward, depending on participant availability and location.

A topic guide was designed and informed by previous relevant research on the psychosocial impact of BC on women [6,21,22]. Participants were asked about their experiences after diagnosis, changes in their personal and social interactions, and the social and interpersonal factors that facilitated or hindered their recovery. The interviewer encouraged participants to express their genuine feelings by actively listening without interruption or judgement. Each interview lasted 30 to 45 min and was audio-recorded and transcribed verbatim to ensure accuracy.

### 2.4. Data Analysis

Collected data were analysed using the framework analysis method, a systematic approach involving five steps: familiarisation with the data, identification of a thematic framework, indexing, charting, and mapping/interpretation [23]. In the initial stage, the researchers familiarised themselves with the data, becoming attuned to early emerging themes within and between participants. Subsequently, we employed both inductive and deductive coding approaches. Inductive coding allowed themes to emerge directly from the data, enabling an in-depth understanding of participants’ experiences. Deductive coding, on the other hand, utilised pre-determined categories informed by the research objectives and the relevant literature. This dual approach ensured that our analysis was both grounded in the study’s focus and capable of recognising key themes derived from a priori considerations, as well as issues that arose during the familiarisation stage [23]. A coding framework was developed, utilising numerical or textual codes to differentiate specific themes. Headings from the thematic framework were utilised to construct a chart summarising the data through a process of abstraction, denoting specific categories from each transcript for each theme. Finally, during the mapping and interpretation stage, the researchers sought patterns and identified associations to gain a comprehensive understanding of all themes and to explain the relationships between them [23].

The initial transcripts were independently coded by the first and second authors, both healthcare professionals experienced in qualitative research. They subsequently met to collaboratively determine the final set of codes. Emergent themes and subthemes were discussed until a consensus was reached. Data collection continued until saturation was achieved, the point at which no new codes or information emerged, rendering further analysis unnecessary [24]. Achieving saturation enhances the credibility and dependability of qualitative findings, as it ensures that the dataset captures a comprehensive range of participants’ experiences and perspectives.

### 2.5. Ethical Approval

This study obtained ethical approval from the Institutional Review Board and Ethics Committee of the SQCCCRC (#CCCRC-06-2022), approved on 5 May 2021, the University Medical City. All participants provided written informed consent before inclusion in the study.

## 3. Results

### 3.1. Characteristics of the Participants

A total of 11 young Omani BC survivors participated in the study. The mean age was 35.0 ± 5.9 years (median: 36.0 years; range: 24–44 years). Two women (18.2%) had been diagnosed at stage II, four (36.3%) at stage III, and five (45.5%) at stage IV. Most were married (54.5%), employed (63.6%), and had graduated from high school or college (72.2%). Over one-third were from the Al-Batinah region (36.4%) and had a total family income exceeding 1000 Omani riyals (54.5%). The vast majority had no family history of BC (72.0%) (Table 1). At the time of the interviews, participants were undergoing various treatment modalities, including chemotherapy, radiotherapy, and hormone therapy.

### 3.2. Findings

Six types of interactions with various groups emerged from the analysis following diagnosis among younger Omani BC survivors: self, children, spouse, family, friends, and society. Table 2 summarises these interaction domains and key findings with regards to resilience.

#### 3.2.1. Self-Interaction

Participants demonstrated a strong sense of acceptance of their cancer diagnosis as a result of their faith, viewing it as part of a divine plan. Their belief in divine guidance, paired with optimism about the available treatments, fostered resilience, allowing them to maintain a calm and hopeful outlook during their journey of treatment and recovery:

*“When I was told that I …* [had] *cancer, I accept*[ed] *it normally because it is the act of God that I should obey. I …* [had] *faith that every disease God has created has a treatment and cure for it. I accepted it normally”*.

(Participant #8, 35 years old)

*“One of my relatives told me that her uncle was diagnosed with cancer, and he was in a bad psychological state. I advised her that he needs to accept it because …* [his] *psychological state is an important factor which will help* [him] *to overcome the disease because the therapy … is strong”*.

(Participant #10, 38 years old)

However, some participants experienced a decline in self-confidence, particularly after treatment. This affected their willingness to socialise and interact with others, leading to introversion and a marked withdrawal from social interactions, often related to changed self-perception or fear of judgment following changes in appearance:

*“After the diagnosis, my self-confidence went down. I was not accepting my appearance as a person. During the treatment period itself, I was very, very strong. After finishing all the treatments, I felt that I was broken and I became an introvert*[ed] *person”*.

(Participant #1, 36 years old)

Despite these challenges, several participants identified positive changes, including increased self-care, attention to detail, spontaneity, and personal growth, indicating belief that the illness had made them stronger. Several participants spoke of prioritising their own comfort and well-being over pleasing others, leading to increased self-care and confidence:

*“I felt that, to be honest, the disease created positives … I felt that I became stronger and I used to focus on small details that I no longer focus on and I became more spontaneous. I started to care about myself more without forcing myself to do things I’m not comfortable* [with] *… for others. I only do what only makes me comfortable and I feel also that I became more confident”*.

(Participant #3, 40 years old)

*“I noticed that I have changed, this disease* [makes] *me strong and it solidifies my personality. I was more dependent on my husband but I started to depend more on myself; now I drop the kids by myself to school. I became more* [in]*dependent”*.

(Participant #7, 33 years old)

#### 3.2.2. Interaction with Children

Participants sought to maintain a sense of normalcy in their relationships with their children, despite their diagnosis. They strived to shield their children from potential worries and the emotional and physical effects of cancer treatment, attempting to ensure that their interactions remained unchanged. Additionally, several participants reported increased intensity in their bond with their children:

*“I did not want to let them* [my children] *feel that there is a difference in our relationship: before and after the diagnosis, it was totally the same. But, deep inside me, I was trying to get closer without letting them notice that”*.

(Participant #1, 36 years old)

*“I became closer to them* [my children]*, I feel that the therapy time made me be closer to them … I have already started the therapy but I …* [haven’t] *told them. They …* [are] *aware that I go to the hospital but they don’t know what I* [am] *doing there”*.

(Participant #10, 38 years old)

*“Even … when I was doing the chemotherapy session*[s]*, I wasn’t going back to my home with my kids. To avoid them seeing me tired … I used to go to my parents’, where I stay for a whole week, and I also used to take the immunity injections in my parents’ house to avoid the* [children] *seeing me”*.

(Participant #5, 24 years old)

Nonetheless, despite attempts to maintain normalcy, the diagnosis often caused emotional distress. Participants expressed anxiety about their prognosis, reflecting the significant impact on their emotional well-being. The belief that a cancer diagnosis signified inevitable death led to preoccupation regarding the emotional and practical challenges that their children would face in their absence:

*“It was a shock for me … I had* [a lot of] *fear, especially* [because] *I do have kids and I was concerned about them. The dominant feeling was distraction, I was too distracted. I did not know what* [would] *happen to me as I know nothing about this disease. All …* [that] *I knew is anyone having cancer will die from it”*.

(Participant #1, 36 years old)

*“I was thinking about my kids and how hard* [it would be] *to pass away and leave them behind; it was difficult”*.

(Participant #10, 38 years old)

Physical changes resulting from the side-effects of cancer treatment also influenced the participants’ interactions with their children during this challenging period. They observed that their children often displayed concern and distress about their health, even though the diagnosis had not been disclosed:

*“My younger daughter once knocked* [on] *the bathroom door while she was in hurry, and …* [during that] *time, I* [had] *started the chemotherapy* [and so] *I was wearing* [a] *cap to cover my hair all the time, even in the house. I opened the door while I was not wearing the cap and she was in shock, to the level that she …* [was unable to] *urinate”*.

(Participant #2, 27 years old)

In such cases, participants made efforts to reassure their children, providing simple and age-appropriate explanations for their changes in appearance:

*“They* [the children] *understood it more when they saw the condition of* [my] *hair later and they started to wonder why you don’t have hair. I was answering that I am sick and* [taking] *medications and it will be growing back after I finish from the therapy. They* [the children] *noticed and I had no choice* [other] *than telling them, and it grew back after I was done from the therapy”.*

(Participant #4, 31 years old)

#### 3.2.3. Interaction with Spouses

Many participants reported that their relationships with their spouses either remained unchanged or became stronger, highlighting the emotional support they received. This support reinforced the participants’ sense of identity and stability, which was crucial for their psychological resilience:

*“He* [my husband] *accepted me as I am. He used to say that I see you* [as] *the same woman as before and I don’t see that anything has changed* [about] *you at all”*.

(Participant #1, 36 years old)

*“My husband is, during the therapy session, he tried to change the environment. We travelled a lot during that time, and I noticed a very positive impact on my psychological state”*.

(Participant #4, 31 years old)

*“We* [my husband and I] *became much closer to each other. We used to have more arguments before; we became better and closer”*.

(Participant #10, 38 years old)

However, the physical and emotional toll of cancer treatment strained intimate relationships for some, leading to feelings of frustration:

*“Honestly, I was feeling, deep inside myself, that all what I wanted from my husband is divorce. With all the medications I was taking and the therapy, I was really exhausted, especially in* [terms of] *the intimacy* [side of the] *relationship because of the dryness … I used to cry and think about divorce … I felt that I am out of the mood … I was worried to put pressure on him, but I passed that period”*.

(Participant #6, 40 years old)

Similarly, some participants reported that the side-effects of cancer treatments adversely impacted their body image, thereby affecting their marital relationships. They experienced feelings of inferiority due to the loss of their breasts, which impacted their sense of femininity and self-worth:

*“In my relationship with my husband, sometimes I feel that I am now different than other women because I eradicated the breast. Sometimes I feel that I am less than the others. But, at the same time, I feel lucky that I survived all of that and I am doing well in my work”*.

(Participant #2, 27 years old)

#### 3.2.4. Interaction with Family

Upon learning of the diagnosis, participants faced varied reactions from family members, including crying, fainting, and collapsing, which highlighted the shock and emotional intensity surrounding their situation. Despite attempts to find a silver lining, the reactions of family members often overwhelmed the participants, causing them to dwell on the gravity of their diagnosis:

*“My sister told them* [my family] *about my diagnosis and the reactions were different: some of them were crying, and there was someone who fainted … I was telling them that definitely this will be having a benefit for me and we don’t know where it is yet. My mother was more than crying, she collapsed, my father was only in tears and my sister … fainted. I was talking to her but she wasn’t responding … I left them and stayed alone in my room. I started to think about everything, and I … cried”*.

(Participant #10, 38 years old)

Other reactions bridged shock to emotional resilience, with participants drawing strength from their family’s support:

*“He* [my brother] *didn’t collapse, it was totally the opposite—he was strong while I collapsed and I was in tears, but at the end of that meeting I decided to accept* [my diagnosis]*, so I can proceed to the treatment”*.

(Participant #3, 40 years old)

*“My sister-in-law took me to another room because I collapsed, and I was in tears wondering why it was me who got this disease. I didn’t want that to happen because I still want to live, because* [I had] *the idea that all people who have cancer are going to die soon”*.

(Participant #6, 40 years old)

In addition to their initial responses, family support provided emotional and practical assistance that helped participants maintain a sense of normalcy and stability during their illness:

*“My father was trying not to let me feel that I was alone or anything has changed. This* [is] *what made me feel that I was strong enough to live a normal life, they* [my family] *didn’t let me feel that I was sick*”.

(Participant #2, 27 years old)

*“They* [my family] *never let me feel anything unusual and they were supporting me”*.

(Participant #6, 40 years old)

Family, relatives, and the tribal community also represented significant sources of psychological, logistical, and financial support, particularly concerning the education and upbringing of children during difficult times. This highlights the participants’ trust in their familial and community support systems:

*“… My family, my relatives, and my tribe, I was supported financially by all of them. For moral support, my family supported me and they took care of my kids to the level that I was not worried about them when it comes to their education and rear* [upbringing]*. I left a newborn who was two months at that time; my mother raised him until he is two years”*.

(Participant #1, 36 years old)

*“I loved their* [my family’s] *support, and it was what I needed the most because I felt like I am living my last days and I will eventually die. I felt that I needed my sister to take care of me because it was only a matter of time and I will not be able to take care of myself and I will be bedridden”*.

(Participant #2, 27 years old)

Shared experiences of supporting one another through hardship also appeared to bring families closer together, even in situations where they had not been particularly closeknit before. Participants noted that this increased closeness, which likely served as a source of comfort and strength, was a significant and valuable outcome of their experience, potentially outweighing their initial lack of intimacy:

*“The best thing was the support I received from the whole family. Although we were always gathering* [before the diagnosis]*, we weren’t close this much to each other”*.

(Participant #10, 38 years old)

*“I was very close with my parents; I do love them a lot and they feel the same, but this connection became stronger after the diagnosis”*.

(Participant #2, 27 years old)

#### 3.2.5. Interaction with Friends

Participants maintained their social connections with friends and colleagues, which helped prevent feelings of isolation even during cancer treatment. As a result, their social lives stayed relatively intact. Friends provided support, encouragement, and advice, and they even offered natural remedies:

*“Even my social life wasn’t affected. My colleagues were visiting me and telling me that if I don’t want to meet people, I can isolate myself because of the disease but I didn’t want to create this atmosphere for myself. It was totally the opposite for me”*.

(Participant #2, 27 years old)

*“My colleagues stood by my side during my therapy journey, they were supporting me and showing that in different forms. Some of them used to bring me natural honey, herbs, and frankincense. They were more than colleagues; I felt that they are my sisters. My colleagues told my close friend once that they can’t visit me often and they were worried that I’ll feel bad about it”*.

(Participant #7, 33 years old)

While some participants initially withdrew from social interactions due to their physical discomfort and mental exhaustion, they eventually resumed their social lives, recognising the importance of staying connected:

*“It* [my relationship with my friends] *was slightly affected. I was the one who started to withdraw but I started to go back to the norm, and I want to because this withdrawal made me feel uncomfortable and I was mentally exhausted at that time. … I truly want to go back to the old days, now I am much better than before, I started also to go out*”.

(Participant #1, 36 years old)

Several participants also highlighted the importance of supportive work environments in managing health challenges, particularly through gestures of support, compassion, and solidarity during difficult times. They valued the discretion shown by colleagues who, upon learning of their diagnosis, refrained from directly inquiring about it and instead demonstrated support by periodically checking on the participant’s well-being:

*“I had to tell the general manger to keep them aware that I will be* [away on] *a long leave* [of absence from work] *and my colleagues knew about it later. They didn’t … dare … to ask me* [directly]*, but they were checking on me from* [one] *time to another”.*

(Participant #3, 40 years old)

#### 3.2.6. Interaction with Society

Some participants opted to keep their cancer diagnosis confidential, sharing it solely with immediate family. This decision stemmed from a desire to minimise distractions during therapy and shield themselves from potential negative attitudes. By confiding only in close family members, they preserved a sense of privacy and control, enabling them to concentrate on treatment without external interference or judgment:

*“When I was diagnosed with this cancer, I stipulated that no one knows about it outside the range of family. I didn’t* [want] *anyone to know about it because I don’t want to be distracted during the therapy period and there is* [are] *people who look at* [these] *things negatively. It was only in the range of my siblings and their spouses, no one …* [else] *knows about it”*.

(Participant #3, 40 years old)

Fearing social stigma, others similarly withdrew from public interactions to avoid judgment or negative attention, recognising the potential for unfavourable scrutiny within the community. They expressed a desire to avoid being the centre of attention, feeling uncomfortable at the potential of stares and judgment from others:

*“I became an introvert after the chemo doses, I didn’t want to talk to anyone to avoid being asked about my disease”*.

(Participant #7, 33 years old)

*“I want it to be confidential and I didn’t want anyone to know or be aware of my situation as I know how the community reacts with it and they give you sometimes a negative message”*.

(Participant #5, 24 years old)

*“What really exhausts me is the hair fall, including the eyebrows and the lashes: whenever I go to shopping malls, I feel that people are having a wandering eye to me, which I don’t like. This was the most difficult to me and the therapy wasn’t difficult as much as the hair loss”*.

(Participant #4, 31 years old)

Participants also emphasised the importance of supporting other cancer patients, particularly through the sharing of common experiences and the significance of maintaining resilience and optimism, which contribute to the effectiveness of cancer treatment. Additionally, they played a role in motivating and encouraging other patients to pursue treatment options available at local hospitals rather than seeking treatment abroad:

*“I wished if I had the chance to meet people who doesn’t have the courage to complete the chemotherapy journey to push them forward, I was telling myself that I wanted to go for the day care unit to achieve this after I finish from the chemotherapy; I wanted to share hope to others”*.

(Participant #11, 37 years old)

*“I also receive messages and calls from people who are recently diagnosed with the disease, and I motivate and push them forward to start the treatment here in the hospital without travelling abroad for it because it is available here”*.

(Participant #7, 33 years old)

## 4. Discussion

To our knowledge, this is the first study to explore the interaction patterns of young Omani BC survivors and the influence of cultural and social factors. The analysis of interactions following a cancer diagnosis among younger Omani BC survivors reveals six key areas: self, children, spouse, family, friends, and society. Participants demonstrated resilience through their faith and optimism, which helped them navigate treatment, despite challenges like declining self-confidence and withdrawal from social interactions. Many sought to maintain normalcy with their children, shielding them from the emotional impact of the diagnosis, while some experienced strengthened bonds. Relationships with spouses remained supportive, though cancer’s physical toll sometimes strained intimacy. Family responses varied from shock to emotional resilience, offering significant support. Friendships helped maintain a sense of normalcy, with participants returning to social activities and appreciating their work environments. Some kept their diagnosis private to avoid stigma, focusing on treatment without external judgment, while others found strength in supporting fellow cancer patients, encouraging local treatment over seeking care abroad.

The findings reveal that the participants experienced significant shifts in their self-perception, relationships, and support systems following their diagnosis. Many displayed a remarkable sense of resilience, actively embracing their faith and accepting their diagnosis as part of a divine plan. This acceptance fostered a sense of inner peace and strengthened their ability to face the challenges posed by cancer. While many participants recognised the importance of maintaining normal relationships, particularly with their children, they also expressed concerns about the emotional impact of their diagnosis on their families. Additionally, the study highlighted the critical role of family support in enhancing emotional resilience, helping participants navigate the complexities of treatment, and fostering a sense of normalcy in their lives.

Participants interacted with their inner selves through a sense of acceptance and faith in the face of their BC diagnosis. For Muslim women, acceptance of a cancer diagnosis is not merely an act of passive resignation; rather, it represents an active trust that *Allah* (God) has a reason for every trial. This belief can foster inner peace amid the uncertainties of their condition, as they have faith that healing or relief will come in time [21]. Furthermore, their connection to their inner selves is deeply rooted in their spiritual outlook, which enhances their ability to face challenges with resilience and trust in divine will, providing both emotional and spiritual comfort [10]. Indeed, despite dealing with substantial physical, emotional, and existential challenges, many of the participants found new inner strength and resilience to adapt and persevere. This transition exemplifies post-traumatic growth, often exhibited by individuals with BC who emerge stronger and better equipped to handle life’s challenges [25]. Nonetheless, for some participants, self-confidence was diminished due to physical changes in their body image, leading to feelings of stigma, fear of judgment, or embarrassment. This, in turn, reduced their sense of self-worth and contributed to social avoidance [6].

Participants also aimed to preserve their relationships with their children after the BC diagnosis, seeking to protect them from potential worries associated with the side-effects of treatment. Emotional responses or physical changes in parents with cancer can induce anxiety, fear, or confusion in children; hence, many parents feel an acute responsibility to shield their children from emotional distress [26]. Indeed, many participants noticed that their children, particularly those who were older, expressed heightened concern and distress about their health, despite the BC diagnosis not being disclosed to them. Additionally, some participants lived with uncertainty and fear regarding their prognosis, feeling acute anxiety about dying and leaving their children behind. They were particularly concerned about the emotional and practical challenges their children would encounter in their absence [6]. Such children may experience significant emotional distress, including difficulties in managing their day-to-day lives and emotional growth, as well as feelings of grief, abandonment, and confusion [27].

The participants in this study felt that the side-effects of cancer treatments, such as reduced libido and breast and hair loss, negatively impacted their body image and relationships with their spouses, leading to feelings of inferiority that affected their sense of femininity and self-worth. Breasts are closely associated with societal perceptions of femininity; thus, losing one or both breasts can significantly alter how women perceive their bodies, often resulting in feelings of disfigurement, shame, and diminished confidence [28]. Several of the participants expressed vulnerability, fearing that their partners would no longer find them attractive or that their marital relationship would change. This fear can lead to anxiety, reduced intimacy, emotional distance, and declining body confidence, adversely affecting marital dynamics and sexual quality of life [29].

Indeed, several participants reported considering divorce due to the pain and distress associated with intimate relations following the side-effects of cancer treatment. Conversely, other participants indicated that their relationships with their husbands remained unchanged or even grew stronger despite the BC diagnosis and treatment side-effects. Mutual resilience and the experience of navigating a life-altering condition like BC together often prompts couples to reassess and strengthen their emotional connection, fostering a deeper bond [30]. Furthermore, the shared challenges of managing treatments and household responsibilities, coping with side-effects, and confronting the uncertainties of cancer can promote a sense of solidarity and deeper commitment, reinforcing the relationship [31].

Participants also noted that the support and care provided by family members bolstered their emotional resilience, enabling them to maintain a semblance of normal life despite their cancer diagnosis. In Oman, cancer patients frequently underscore the critical role of familial support in enhancing their emotional strength and sustaining a sense of normalcy during cancer treatment [32]. Omani society, like many Arab cultures, places a high value on extended family and tribal systems, viewing family as central to personal and social identity. This cultural context enhances both the mental and emotional well-being of cancer patients [33]. The emphasis on communal values, religious faith, and familial responsibility further reinforces the family’s role in a cancer patient’s journey, promoting resilience and improving quality of life [6]. Family support also offers a sense of belonging and protection, aiding patients in coping with the physical and emotional challenges of cancer treatment, while also providing security [33]. Additionally, the presence of family members can mitigate negative psychological effects, such as anxiety, depression, and stress, by fostering a positive outlook and acting as a protective factor against severe psychological distress [34]. Social interactions with family members also contribute to feelings of happiness, self-worth, and connection, which are crucial for overall psychological well-being [35].

In our study, support for cancer patients extended beyond immediate family members to include extended relatives, friends, and the broader tribal community in Oman. These networks provided essential psychological, social, and financial assistance, including support for children’s education and upbringing during times of hardship and preventing feelings of isolation. During periods of illness, friends and tribal members often step in to ensure that the patient’s family, particularly their children, are well-supported [36]. Islamic values central to Omani culture emphasise the importance of assisting those in need, especially relatives and neighbours. Charitable acts encouraged by Islamic teachings extend to providing financial or emotional support to cancer patients within the family or tribe, which help to reduce anxiety and stress while fostering emotional resilience [37].

Nonetheless, despite efforts by family members to remain composed and supportive, participants in the study reported varied emotional reactions from their relatives when sharing their cancer diagnosis, including shock, crying, fainting, and even collapsing. Family members often experience significant stress, grief, and fear, which can manifest as physical reactions tied to an overwhelming psychological burden associated with a cancer diagnosis. These responses are a natural coping mechanism in response to the life-altering implications of the diagnosis [15]. The fear of losing a loved one and the inherent uncertainty of the disease prognosis often contribute to these intense emotional reactions, which reflect a broader desire to protect and support the patient [21].

Some participants expressed fears of social stigma related to their cancer diagnosis, prompting them to conceal their condition from everyone except immediate family and spouses. This decision allowed them to avoid negative interactions and judgments from those they believed held unfavourable views, enabling them to focus on their health without external interference. In many Arab countries, including Oman, cancer often has negative connotations, such as fatalism and shame, causing patients to worry about being perceived as weak or burdensome [38]. Furthermore, social stigma can significantly impact the psychological well-being of cancer patients, leading to increased anxiety and depression, which hinders their ability to cope with the challenges of treatment and recovery [6]. Conversely, cancer patients in other studies who perceive a lack of support from their community often report heightened feelings of isolation, emotional distress, and decreased motivation to engage in self-care or adhere to treatment, further exacerbating their overall sense of distress [39].

### Limitations

This study has limitations. First, while this study provides valuable insights, it is important to acknowledge its limitations. Although generalisability of qualitative findings is typically not expected, it should be noted that our participants were recruited from a major oncology centre in Oman, which serves patients from all over the country. Therefore, while the findings provide valuable insights into the experiences of young Omani BC survivors, they may not be applicable to other countries with differing cultural contexts or healthcare systems. Indeed, qualitative research is not aimed at the generalisability of findings but rather at providing in-depth insights and understanding of the experiences, perspectives, and contexts specific to the participants under study. Second, while we documented and reported the stage of the disease at the time of data collection, the time since diagnosis was not systematically collected. This omission limits our ability to account for variations in patients’ experiences, which may evolve over time after a cancer diagnosis. Finally, another limitation of the study is the presentation of sociodemographic characteristics in a summarised format rather than on a case-by-case basis. While a case-by-case presentation could provide a more individualised context for the quotes and narratives shared, the current study design did not systematically link specific quotes to detailed participant profiles.

## 5. Conclusions

In conclusion, we identified six key domains of interaction among young Omani women following a BC diagnosis: self, children, spouse, family, friends, and society. The majority demonstrated strong faith and acceptance, viewing their illness as part of a divine plan, which promoted optimism and resilience. However, a subset experienced diminished self-confidence, particularly after treatment, leading to social withdrawal due to altered body image. Despite these challenges, many women reported personal growth, enhanced self-care, and a renewed focus on their own well-being.

Interactions with children were characterised by efforts to preserve a sense of normalcy, although concerns about the future and their children’s well-being were prevalent. Emotional support from spouses generally reinforced marital relationships, despite ongoing challenges related to sexual intimacy and body image. Family members played a critical role in providing emotional, psychological, and financial support, contributing to a sense of stability and normalcy. Support from friends and colleagues was typically positive; however, societal stigma led some participants to conceal their diagnosis.

This study underscores the necessity of psychosocial interventions aimed at enhancing self-confidence and facilitating social reintegration. Parental guidance programs are essential to support effective communication with children while maintaining emotional stability. Couples therapy is recommended to address intimacy and body image concerns, providing support for both BC patients and their partners as they navigate these challenges. A family-centred approach to care is vital for offering emotional support and strengthening familial bonds. Community-based education programs should focus on reducing stigma, thereby empowering patients to disclose their diagnosis without fear of judgment. Additionally, workplace support programs are critical for ensuring emotional well-being and privacy. Finally, peer support networks can play a pivotal role in fostering resilience by encouraging survivors to share their experiences.

Finally, future research is needed to explore tailored psychosocial interventions, parental guidance programs, and couples therapy to address self-confidence, body image, family communication, and intimacy challenges among young BC survivors. Studies should also examine family-centred care models, community education to reduce stigma, workplace support policies, and peer networks to foster resilience. These efforts will inform culturally sensitive, evidence-based strategies to support survivors’ psychosocial well-being.

## Figures and Tables

**Table 1:** Self-reported sociodemographic characteristics of the participants (N = 11).

Characteristic	N (%)
**Age (years)**	
Range	24–44
Mean ± SD	35.0 ± 5.9
Median	36
**Marital status**	
Single	4 (36.4)
Married	6 (54.5)
Divorced/widowed	1 (91)
**Education level**	
High school	1 (9.1)
College/university	8 (72.7)
Postgraduate	2 (18.2)
**Employment status**	
Employed	7 (63.6)
Unemployed/housewife	4 (36.4)
**Monthly family income (OMR)**	
≤1000	5 (45.5)
1000–2000	5 (45.5)
>2000	1 (9.1)
**Stage of BC at diagnosis**	
II	4 (36.4)
III	2 (18.1)
IV	1 (9.1)
Unknown	4 (36.4)

Abbreviations: BC = breast cancer, OMR = Omani riyal, SD = standard deviation.

**Table 2:** Summary of findings of interaction among young Omani breast cancer survivors (N = 11).

Interaction Domain	Type of Resilience	
	Positive	Negative
Self	Belief in divine ordination, leading to obedient acceptance. Strengthened faith in God. Enhanced self-care and confidence, with a greater focus on personal comfort and well-being over pleasing others.	Reduced self-confidence due to the side-effects of cancer treatments and decreased social interaction. Distorted self-perception driven by fear of judgment.
Children	Maintenance of normal relationships despite challenges. Increased attention to loved ones, driven by anxiety about dying and leaving children behind.	Worry when children express concern and distress about the participant’s health due to changes in appearance caused by treatment side-effects.
Spouse	Maintenance of normal relationships, despite challenges. Strengthening of relationship and marital bond during illness.	Worry about burdening spouse. Contemplating divorce due to treatment side-effects. Feelings of inferiority, loss of femininity, and diminished self-worth due to changes in appearance caused by treatment side-effects.
Family	Strength and support during difficult times. Maintenance of sense of normalcy. Strengthening of relationships and becoming closer during illness. Social and financial support.	Shock and intense emotions following the diagnosis.
Friends	Support and encouragement to combat social isolation. Empathy and concern within the workplace.	Withdrawal from social interactions due to mental exhaustion and discomfort.
Society	Motivation and encouragement to pursue treatment within the country rather than abroad.	Hiding the diagnosis to avoid potential negative attitudes, external interference, or judgment. Fear of social stigma or criticism due to the diagnosis.

## Data Availability

The datasets are unavailable online due to privacy or ethical restrictions. Data are available from the corresponding author upon reasonable request.
